# The Electrical Response of Real Dielectrics: Using the Voltage Ramp Method as a Straightforward Diagnostic Tool for Polymeric Composites

**DOI:** 10.3390/ma15113829

**Published:** 2022-05-27

**Authors:** Paolo Vitulo, Michele Zanoletti, Riccardo Morina, Daniele Callegari, Eliana Quartarone, Riccardo Viola, Davide Comoretto, Sergio Dulio, Piercarlo Mustarelli, Maddalena Patrini

**Affiliations:** 1 Dipartimento di Fisica, Università degli Studi di Pavia, Via A. Bassi 6, 27100 Pavia, Italy; paolo.vitulo@unipv.it (P.V.); michele.zanoletti01@universitadipavia.it (M.Z.); 2 Dipartimento di Scienza dei Materiali, Università Degli Studi di Milano Bicocca, Via R. Cozzi 55, 20125 Milano, Italy; r.morina@campus.unimib.it (R.M.); piercarlo.mustarelli@unimib.it (P.M.); 3 Dipartimento di Chimica, Università Degli Studi di Pavia, Via T. Taramelli 12, 27100 Pavia, Italy; daniele.callegari01@universitadipavia.it (D.C.); eliana.quartarone@unipv.it (E.Q.); 4 Atom S.p.A., Via Morosini 6, 27029 Vigevano, Italy; riccardo.viola@atom.it (R.V.); segio.dulio@atom-lab.org (S.D.); 5 Dipartimento di Chimica e Chimica Industriale, Università Degli Studi di Genova, Via Dodecaneso 31, 16146 Genova, Italy; davide.comoretto@unige.it

**Keywords:** dielectrics, electrical response, polymeric composites, lumped circuits modeling

## Abstract

An experimental method exploiting the capacitive response of most materials is here revised. The procedure called the “Voltage Ramp Method” (VRM) is based on applying proper voltage ramp cycles over time and measuring electrical current intensity flowing through the material sample. In the case of an ideal capacitor, a current plateau should be easily measured, and the capacitance value precisely determined. However, most media, e.g., semiconductors and insulating polymers, show dielectric absorption and hence electric leakage effects. Therefore, the VRM method allows simultaneous determination of their equivalent capacitance and resistance. Some case studies are discussed as concerning the application of VRM to both standard and actual media. A figure of merit of the method is the percentage difference between 2.5% and 1.5% with respect to the nominal values of a commercial capacitor and resistor, respectively. The simulation modeling of the material electrical response is compared to the experimental data also on polymer nanocomposites suitable for energy harvesting.

## 1. Introduction

The experimental determination of the electrical response of dielectric materials is generally not trivial and requires specific ASTM standard D257 [1] in order to record reliable data. This is particularly critical when dealing with high impedance media, such as polymers and their nanocomposites, where phase transitions, conformational changes, and dipole orientation induce low frequency absorption [2,3]. Major difficulties arise from polarization effects in the material when subjected to an external electric field and to any shunt capacity that may appear in the experimental setup due to its finite input impedance, anisotropy, and electrode deposition. Usually, volume or surface resistivity and capacitance are the main parameters to be determined, and the so-called “step response” or “alternate polarity” [4] methods are the most used in the experiments. 

In traditional investigation methods on the DC electrical response of a material, a voltage stimulus is applied across a sample portion by means of conducting electrodes, and the current intensity flowing through them is measured by the insertion of an electrometer or a picoammeter along the circuit. This setup configures the material under test as the dielectric filler of a capacitor. The simplest way to measure the material response would be to apply the input voltage stimulus as a step. The expected output current intensity would exponentially decrease according to the relaxation time (s) of the sample, thus allowing for a microscopic investigation of material properties. After a characteristic timelapse, a current plateau is reached, and its value is used according to Ohm’s law with the voltage step value to calculate the static resistance value. The geometric sample parameters then allow the calculation of the material’s resistivity. This method is called “step response”. When the resistivity of the sample is remarkably high, however, a slightly different method foresees the application of several step voltages of alternate polarity. This procedure, called the “alternate polarity” method, allows one to discard the effect of background currents which may arise due to electrical charges stored in the sample, a typical problem in polymers. 

A settling time is mandatory to achieve a reliable current reading in the step response method; alternate polarity eliminates any spurious effect, which should add an incorrect constant current contribution to the measured effect, which is the most time consuming for highly resistive materials. In both cases, the steepness of the input voltage stimulus introduces an abruptly high current intensity in the system (due to the capacitive response of the dielectric material under test) that then decays exponentially, driven by time factors characteristic of the material under test.

A third method of exploiting the main capacitive behavior of most materials is here revised. When a constant voltage ramp over time is applied across an ideal capacitor, a constant current intensity flowing through it is observed; the capacitance value is then extracted from the ratio of the constant current value to that of the voltage ramp. This procedure is here termed the “Voltage Ramp Method—VRM”. If this were the only case, this procedure would not be of much interest. However, the electric behavior of most materials is not only capacitive. In fact, most media, particularly polymers, exhibit dielectric absorption and hence electric leakages through them. In this case, the VRM method allows measurement of the static dielectric response of the material. 

An example of application of the voltage ramp concept is for dielectric materials adopted in power transformers, where voltages of the order of tens of kilovolts (with ramps of 1 kV/min) are needed to check the quality of the insulation. However, in this case, only the nonlinearity of the measured current during the ramp is checked and possibly compared with similar plots obtained with the original (new) material [5] so as to evaluate material aging or damage. Moreover, a quite different method used for the same purpose involves applying voltage steps on the order of 1 kV through the material for a certain time and then short-circuiting the material itself to measure the so-called recovery voltage after a discharge phase [6,7]. 

More recently, the ramp method has been applied in electrophysiology, i.e., in the study of ion channels of cell membranes, with voltages of hundreds of mV and ramps of hundreds of mV/s [8]. In the following study, this method is applied with voltages of the order of tens of volts and ramps of a few V/s to insulating and high-k materials. In summary, by reviewing the literature’s methods in the wide material science field (from good conductors to semiconductors to insulators), one can find: on one hand, a few well-established commercial systems to be chosen depending on the order of magnitude of resistance (e.g., alternate Polarity method); and, on the other hand, analytical models for complex impedance versus frequency which are difficult to implement and specific to a material category (e.g., space-charge-limited model for polymers [9]). We believe that our method is somehow intermediate, being simple enough to be implemented and at the same time allowing us to obtain the DC resistance and capacitance values of real samples through phenomenological lumped circuit modelling.

In this article, we apply the method to polymer nanocomposites to be used as energy harvesters, where the VRM method is also useful as a diagnostic tool when comparing materials after aging and fatigue cycles with respect to the original ones. Results are compared to those achieved with suitable standard methods. 

## 2. Materials and Methods

**Figure 1:**
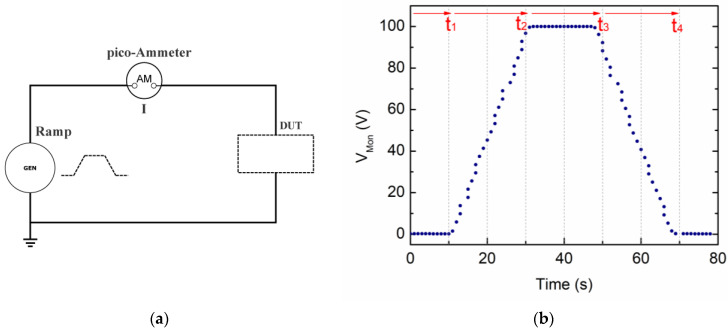
(**a**) Schematic circuit of the experimental VRM setup; (**b**) input voltage cycle used for VRM tests as directly monitored by the HV Power Supply. The ramp-up and ramp-down rates were set to 5 V/s and maximum voltage was set to 100 V. Each measurement cycle lasted approximately 100 s.

In the experimental setup, we adopted an external voltage generator (CAEN R1470ET 4 channel HV Power Supply by CAEN SpA, Viareggio, Italy) and a Keithley 6517B picoammeter with a Keithley 8009 cell resistivity test fixture (with 54 mm diameter electrodes and a guard ring) by Tektronix, Inc. (Beaverton, OR, USA). A simple circuit is realized (Figure 1a) in which the picoammeter measures the output current intensity from the sample (device under test—DUT) placed in the cell and subjected to the voltage cycle stimulus.

A voltage ramp from a generator (typically V/s according to the material specie) is applied to the device under test for the time interval necessary to reach a voltage plateau; then, a constant voltage value is maintained for a second time interval; and, finally, an opposite-slope voltage ramp is applied down to zero voltage. A picoammeter is inserted in series into the material under test to measure the intensity current profile along the voltage cycle. The absence of a strong steepness in the time domain corresponds to the absence of higher frequencies in the fast Fourier transform (FFT) content of the stimulus signal and hence a reduction of initial spikes in the measured current. Figure 1b shows the typical input voltage function as monitored by the HV supply itself. This can be recursively applied to the studied sample, with the polarity being chosen by the user as preferred. The leading and the trailing ramps do not necessarily have the same rate.

During the VRM cycle, depending on the resistive and capacitive properties of the material under investigation, the well-known phenomena of accumulation/release of charge and dispersion of energy occur [10]. These are normally described by the standard equations of electrical circuits and current intensity evolution must be calculated according to the equivalent circuit model of the material under examination.

Following the V_Mon_ voltage sequence of Figure 1b, we divide the VRM cycle into four time intervals which will be useful in the following to analyze the different cases: baseline (from 0 to *t*_1_), increasing ramp (from *t*_1_ to *t*_2_), flat top (from *t*_2_ to *t*_3_), and decreasing ramp (from *t*_3_ to *t*_4_). As a general rule, we write appropriate equations for the current intensity in each time interval, as reported in Appendix A. Depending on the specific material under test—and its equivalent circuit model—we will perform a fit procedure to the experimental data with some free parameter values to be determined. Appropriate initial conditions are set to have analytical continuity at the boundaries *t*_2_ and *t*_3_.

In the following, electrical response simulations and VRM modelling applied to ideal circuits are presented with the aim to prove the potentiality of the VRM technique. Electrical response simulations were performed using LTspice^TM^ software by Analog Device, Inc. (Norwood, MA, USA) [11]. Then, VRM examples with actual materials and *I(t)* experimental measurements are presented as case studies in the Results section. First, to reproduce an ideal R-C behavior, we used bare electronic components: an 8.2 nF capacitor (20% tolerance) and 1 GOhm resistor (1% tolerance). The actual materials we used are: a high-density polytetrafluoroethylene (HD PTFE) slice, 2 mm thick, from Goodfellow GmbH (Hamburg, Germany) as a quasi-ideal insulator; a quartz slice sample, 540 micron thick, as an example of insulator with leakages; a polymer–ceramic composite TPU (Estane 58887, Lubrizol, OH, USA), CaCu_3_Ti_4_O_12_ at 50:50 vol% [3]—hereafter TPU-CCTO—as an example of material with dielectric relaxation behavior.

### 2.1. VRM Modelling

#### 2.1.1. A Single Capacitor as the Ideal Insulator

**Figure 2:**
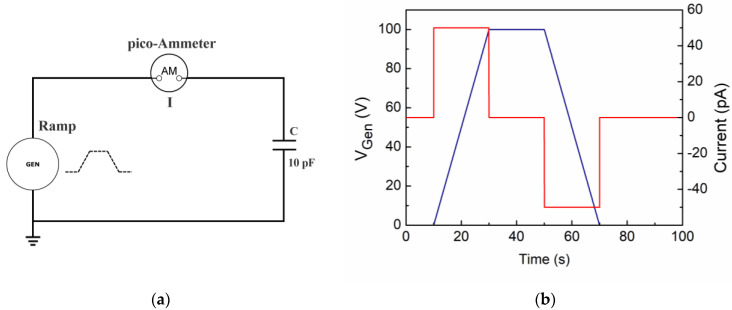
A 5 V/s voltage ramp is applied to a commercial capacitance *C* = 10 pF: (**a**) electrical circuit schematic; (**b**) the applied voltage stimulus (blue line—left scale) is shown. The output current (red line—right scale) shows the expected behavior for an ideal capacitor (see text).

Figure 2a shows the electrical circuit scheme and its simulated output when a VRM cycle from a voltage generator is applied to an ideal capacitance C_DUT_ = 10 pF, then simulating a material with electrical energy storage capability without losses. The expected output current profile is shown in Figure 2b (red line—right scale); the material capacitance is calculated as the ratio of the current intensity plateau (50 pA) to the voltage ramp rate adopted (5 V/s). We note that this should be considered a static (DC) capacitance value to be compared to that determined by LCR multimeters at extremely low frequency values.

#### 2.1.2. A Dielectric with Resistive Losses 

**Figure 3:**
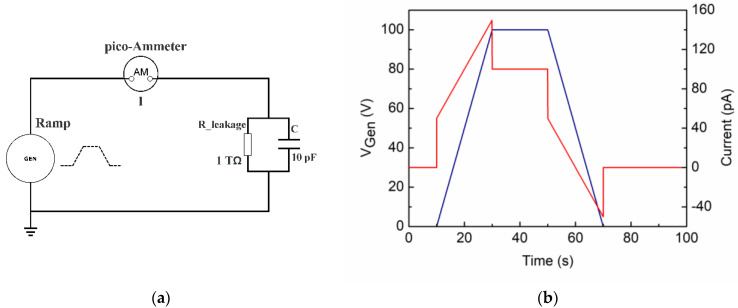
(**a**) Simulated circuit with a capacitor (10 pF) in parallel to a resistor (10^12^ Ohm); a 50-Ohm resistor (not shown) was also added to simulate the output impedance of the generator. (**b**) The applied voltage ramps up/dw (+/− 5 V/s) are shown in blue (left scale), and the simulated current intensity is shown in red (right scale).

In most cases, however, when a voltage is applied across a material, a leakage current appears due to its dielectric losses; in an ideal circuit scheme, the effect can be introduced by a resistor (e.g., *R* = 10^12^ Ohm) in parallel to the test capacitor (Figure 3a). 

Figure 3b shows the result of the electrical simulation of the circuit shown in Figure 3a. In particular, the generated VRM cycle is shown (blue line—left scale) with the usual increasing and decreasing ramp (+/− 5V/s) and a plateau at 100 V. The corresponding current supplied by the simulation (red line—right scale) that passes through the test device is no longer constant; once the value of 50 pA is reached (the value that the capacitor only would maintain throughout the ramp), a constant rise begins which ends when the generator reaches the end voltage (100 V). This current ramp is due to the nonideality of the capacitor and is given by the ohmic current passing across the resistor in parallel. 

The ratio between the value of the generator ramp rate (5 V/s) and that of the current (5 × 10^−12^ A/s) is precisely the resistance R (10^12^ Ohm).

## 3. Results and Discussion

### 3.1. Quasi-Ideal Insulator Material

**Figure 4:**
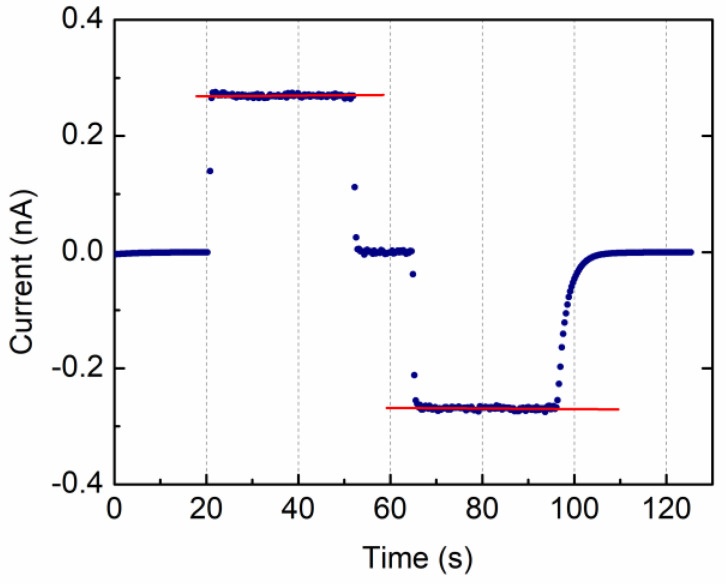
VRM applied to a slice of HD PTFE 2 mm thick. A 10 V/s voltage ramp is applied up to a flat top of 500 V; the output current (blue marks) shows the expected behavior for a real capacitor. Linear fits (red lines) to the top and bottom flat current intensity plateau are also shown.

We adopted the 2 mm thick HD PTFE sample as an ideal insulator prototype. A VRM cycle with a voltage ramp rate of 10 V/s and a flat top of 500 V has been applied to the material in the cell. Figure 4 shows the measured current intensity as a function of time during the VRM cycle. The experimental data (blue points) show a behavior similar to the one expected for the ideal capacitor, except for the steps that are no more perfectly vertical. They are characterized by a weak exponential trend, as simulated and expected from a real system. Best-fits to the flat current tops (red lines) give a capacitance value *C* = 7.98 ± 0.01 pF (baseline offset subtracted). This allows us to calculate the static relative permittivity ε_r_ through the formula *C* = ε_0_ ε_r_ S/t, where S is the sample area and t its thickness; it results ε_r_ =1.8, i.e., within 10% from the nominal value of 1.9–2.0 reported in the literature [12,13]. However, as aforementioned, the value obtained with the VRM technique must be considered as a DC or “zero frequency” value.

### 3.2. Insulator Materials with Leakage

**Figure 5:**
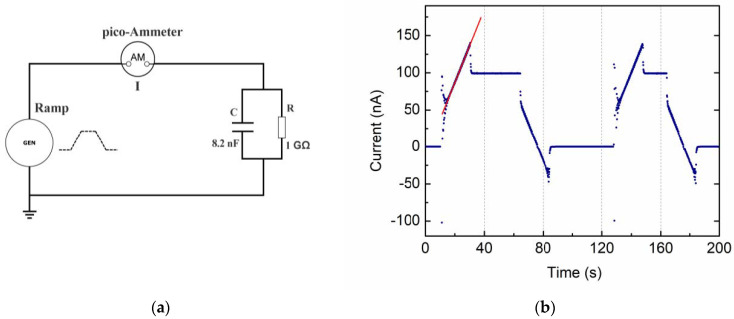
VRM method applied to a circuit with a standard 8.2 nF capacitor in parallel to 1 GOhm resistor; two VRM cycles were applied: (**a**) schematic of the measurement setup; (**b**) current intensity output vs. time as measured by the picoammeter (blue points) and linear fit regression (red line).

To test the VRM performance on a real dielectric with losses, we used as a DUT a standard 8.2 nF capacitor (20% tolerance) connected in parallel to a 1 GOhm resistor (1% tolerance). Here, we applied the VRM cycling twice. The measured current intensity versus time is shown in Figure 5b (blue points) along with fit results to the linear current trends (red line) corresponding to the four ramps of the VRM stimulus (see Table 1). Initial and final damping oscillations are also visible and are due to both impedance mismatch and cabling that introduce additional inductance. Linear fits to the current ramps were used to extrapolate the resistance value that is *R* = 1.014 ± 0.001 GOhm. The capacitance value can be inferred from the ratio of the current step (*I(t*_2_*)*–*I(t*_3_*)*) to the voltage ramp. The C value of 8.02 ± 0.22 nF is within 2.5% of the nominal one and well inside the capacitance tolerance.

**Figure 6:**
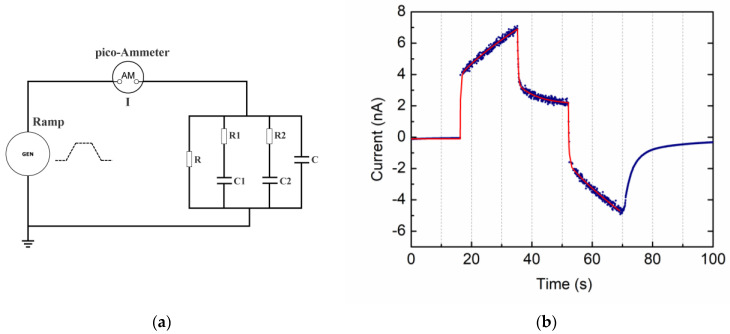
VRM method applied to a 540 μm thick quartz slice. The input voltage ramp-up/down is 25 V/s and the top value is 500 V; (**a**) schematic of the electrical circuit used for the analytical model. Two relaxation times have been added to the standard R-C circuit in order to take into account the current decrease after the flat top voltage is reached; (**b**) the shape of the current output (blue marks) shows a loss due to the finite resistance of the material during the voltage ramp-up and ramp-down. An analytical function (red line) for the current has been used to fit the data.

A real case of dielectric insulator with leakages is reproduced by a quartz slice. We applied the VRM method to this material to test the above electrical behavior. Figure 6b shows the corresponding output current intensity (blue points) vs. time as measured by the pico-ammeter. If no losses were present, we would have observed a constant current due to the capacitance only, as in Figure 4. We note that the current output shape is not exactly that of Figure 5b, but all the features are present. Starting at about 16 s, the voltage from the power supply is increasing with a constant rate (25 V/s) up to the maximum of 500 V at about 36 s. The corresponding current ramp from about 4 nA to about 7 nA is due to the resistance R in parallel to the total capacitance. Moreover, when the voltage plateau is reached and, correspondingly, the maximum current is achieved, we do not observe a steep decrease of the current as in the case of Figure 5b. Rather, a relaxation behavior is observed. We modelled such a behavior as in the circuit of Figure 6a by adding two additional RC branches (relaxation times *τ_1_* = 6.75 s, *τ_2_* = 0.25 s).

**Figure 7:**
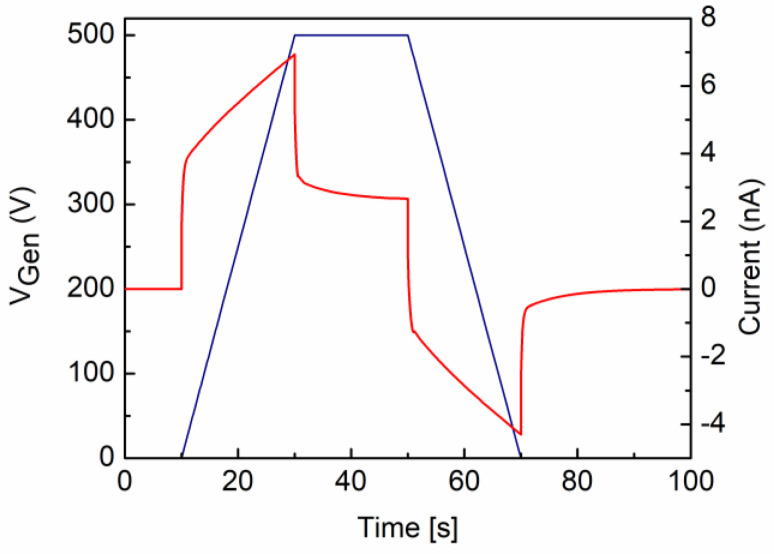
Simulated output (LTspice^TM^) for a VRM cycle applied to the circuit of Figure 6a with parameters obtained from the best fit to experimental data with the equivalent circuit model. The expected output current intensity is shown in red (right scale); the voltage input function is shown in blue (left scale).

The analytical model for the current intensity has been applied and a best-fit procedure (red line) to the experimental data has been performed to obtain the total capacitance and the value of the leakage resistance: C_tot_ = 173.4 ± 32.3 pF, *R* = 189.8 ± 13.0 GOhm. From the capacitance, the value ε_r_ = 4.6 was assigned to the quartz under test to be compared with that which was estimated by the LCR impedance measurement at 20 Hz (ε_LCR_ = 4.62). The alternate polarity method output on the same sample was used to compare this outcome, giving R_AP_ = 182.8 ± 4.6 GOhm with a 3.8% difference in the VRM result. Nonetheless, as a further comparison, the VRM cycle simulation of the equivalent circuit of Figure 6a with parameter values from the fit has been performed. The output for current intensity (red line—right scale) is shown in Figure 7 for an input voltage function (blue line—left scale) identical to that of the test (500 V, 25 V/s). The agreement between the measured and simulated current spectra is very good.

### 3.3. Ceramic–Polymer Composites

**Figure 8:**
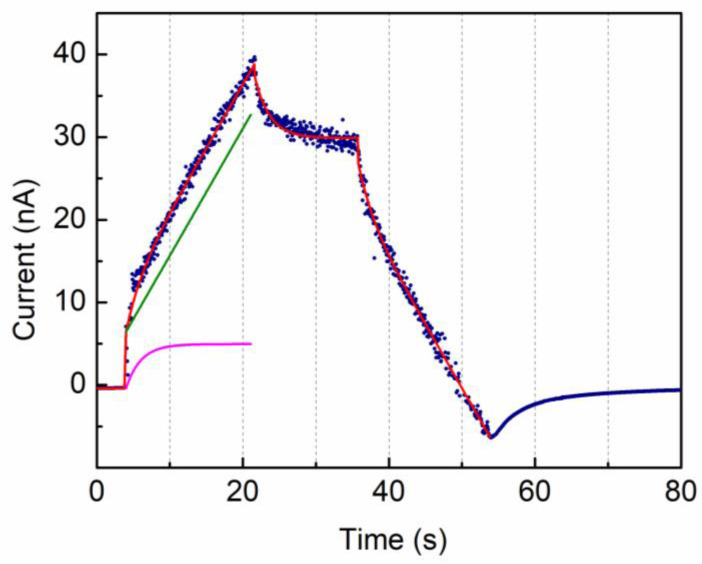
Example of VRM method applied to a 100 μm thick TPU-CCTO composite. The input voltage ramp-up/down was 5 V/s, and the flat top voltage 100 V. Experimental data (blue marks) are outputs from the picoammeter. The red solid curve corresponds to the best fit made to the data with the analytical curve representing the electrical model of Figure 6a. Referring to that scheme, the solid green curve represents the contribution due to the current through R and C, while the solid purple curve shows the contribution of the currents flowing through C1 and C2.

We applied the same model in testing some polymeric composites of TPU-CCTO with the VRM technique. Due to the thickness of the samples (hundreds of μm), the flat top of the input voltage function was limited to 100 V with a typical ramp-up/down of 5 V/s. A VRM cycle example is shown in Figure 8, where the TPU-CCTO current (blue marks) is shown together with the analytical curve (red line) of best fit. We underline that only raw data are shown, and no attempts have been made to improve noise or acquisition parameters (i.e., picoammeter sampling time). Taking as reference the equivalent circuit model of Figure 6a, an initial current step (around 5 s) corresponding to the onset of the voltage ramp is measured due to the capacitance C value of the material. The following current ramp is due to the sum contribution of the leakage resistance R (green curve) and the equivalent capacitors C1 and C2 (purple curve). At the flat top of the input voltage (starting at about 23 s) the current through the capacitance C stops and the remaining currents start decreasing, driven by the relaxation times, up to the point where the negative ramp of the voltage stimulus begins. A decrease in the current which is similar to the increasing one begins. After about 55 s, the input voltage stimulus is turned off and the system returns to the initial status.

For the sake of comparison, the alternate polarity method on the same sample gave a percentage difference within 16% for the value of R (R_VRM_ = 3.43 ± 0.02 GOhm, R_AltPol_ = 2.96 ± 0.07 GOhm).

The VRM method is then demonstrated to be particularly suitable for dielectrics with resistive losses, and in general for polymeric materials, when a phenomenological modelling of the material electrical response is needed without having to resort to an intensive study of the intrinsic polarization phenomena. Such a study would require specific experimental and more complex analytical treatments subject to tentative physicochemical process hypotheses.

## 4. Conclusions

An experimental technique based on a voltage stimulus applied across a material (VRM) is presented and proposed as a simple and powerful method for investigating electrical properties of dielectric media. With respect to the usual methods (alternate polarity or step response) in the present case, a ramp voltage cycle is applied. Beyond the ease of experimental procedure, the advantage here is to avoid unnecessary initial high-level currents though the sample during the steep voltage transition, resulting in high settling times. Additionally, an added value of the VRM method is the possibility of determining both the total resistance and capacitance of the sample according to the best-fitted equivalent circuit model set up to reproduce the sample’s electrical response. The voltage ramp method potentiality should be improved in a multilayer sample structure, where the comparison between simulated and experimental intensity current trends over a cycle should provide the reason for the quality of surface and interface electrical response of materials and junctions.

## Figures and Tables

**Table 1:** Fit results of a linear regression to the four ramps of the current VRM output of Figure 5b.

	Slope (nA/s)	Slope error (nA/s)	Offset (nA)	Offset Error (nA)	Degrees of Freedom	$R^{2}\;\mathrm{Fit}\;\mathrm{Goodness}$	R (GOhm)
Fit 1	4.92	0.02	−11.63	0.46	108	0.998	1.015
Fit 2	−4.92	0.02	375.78	1.25	99	0.999	1.015
Fit 3	4.93	0.03	−590.57	3.79	91	0.997	1.013
Fit 4	−4.94	0.02	868.66	3.16	100	0.999	1.013

**Figure A1:**
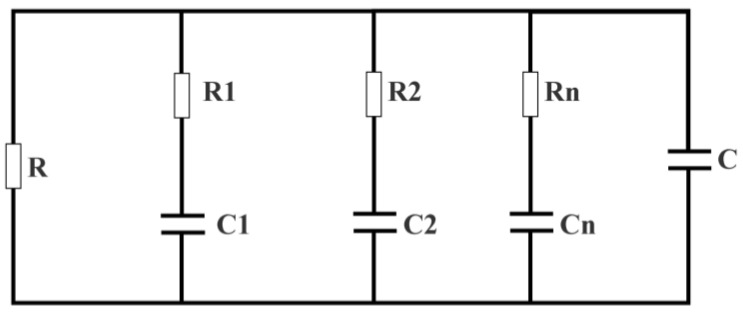
Generic electrical model for a material with n relaxation times. The input voltage stimulus is applied at time *t*_1_ and starts a ramp with rate *k* (V/s) until *t*_2_. A constant value *HV* is held for the time interval *t*_3_ − *t*_2_. A ramp with rate −*k* (V/s) follows for the time window *t*_4_ − *t*_3_. At *t*_4_, the input stimulus ends, and the system returns to its unbiased status.

## Data Availability

The data that support the findings of this study are available within the article and from the corresponding author upon reasonable request.

## Appendix A

Here we report on the analytical equations for the total current intensity *I(t)* used to fit the experimental VRM data from a generic material in the approximation of n-relaxation times, as shown in Figure A1. As already recalled, the resistor *R* and the capacitor *C* account for the DC current leakage through the material and for high frequencies leakage, respectively.

In the time window *t* < *t*_1_, the baseline current value is calculated as the arithmetic average of the measured values. At this time, the increasing voltage ramp has not started yet.

In the time window *t*_1_ ≤ *t* < *t*_2_—Equation (A2)—the input voltage is a linear function of time with slope *k* (V/s); the total current is the sum of the currents through all the n branches. The first term of the equation comes from the usual relation between the current through a capacitance and the time derivative of the voltage across it (in this case only *k*). The second term is the current through the leakage resistance *R* with the direct application of Ohm’s law, the input voltage being *V* = *k* (*t*−*t*_1_). The summation term considers each branch of the circuit with a resistor *Ri* in series to a capacitor Ci. Each branch manages a relaxation time *τi* = *Ri Ci*, and the corresponding current saturates over time according to it. The last term is the baseline.

At the flat top *t*_2_ ≤ *t* < *t*_3_—Equation (A3)—the voltage is constant and the current flow through the capacitance *C* stops. Only the current intensity through the resistor *R* and through each branch remains. The first term again corresponds to Ohm’s law at the constant value *HV*, while the summation of exponential terms considers the relaxation times of each branch. After some time (that in the usual step response method corresponds to the settling time), the total current is *HV*/*R*.

Finally, in the last time window *t*_3_ ≤ *t* < *t*_4_—Equation (A4)—the input stimulus decreases with a constant voltage ramp (in this case −*k*, but its absolute value should be different from the initial value). In the last equation, the terms are similar to the second equation, but opposite in sign and taking into account the different initial time (*t*_3_ instead of *t*_2_).

{(A1)I(t) =                    baseline t<t1(A2)I(t)=kC+kR(t−t1)+∑i=1nkCi(1−e−t−t1τi) t1≤t<t2(A3)I(t)= HVR+∑i=1nAie−t−t2τi t2≤t<t3(A4)I(t)=−kC−kR(t−t3)−∑i=1nkCi(1−e−t−t3τi)+HVR t3≤t<t4

Free parameters of the fits to the experimental *I(t)* values are *R*, *C*, *C_i_*, and *τ_i_*. We observe that in the second equation, if the leading ramp lasts long enough, the exponential terms in the summation can be neglected and a simple linear fit to the *I(t)* data gives both *R* and the total capacitance *C* + $\Sigma_{i=1}^{n}\;C_{i}$ of the sample.
